# An Indigenous *Pseudomonas chlororaphis*-like Isolate M6 Is Associated with Reduced Cotton Damping-Off and Partial Redox–Phenylpropanoid Molecular Profiles

**DOI:** 10.3390/plants15152305

**Published:** 2026-07-27

**Authors:** Yingming Wei, Qiuyue Zhao, Xiaolei Cao, Quanling Zhang, Danni Gu, Nan Qi, Zhaoqun Yao, Sifeng Zhao

**Affiliations:** Key Laboratory at the Universities of Xinjiang Uygur Autonomous Region for Oasis Agricultural Pest Management and Plant Protection Resource Utilization, Agriculture College, Shihezi University, Shihezi 832003, China; weiyingming@stu.shzu.edu.cn (Y.W.); zhaoqiuyue1@stu.shzu.edu.cn (Q.Z.); caoxiaolei@shzu.edu.cn (X.C.); 15276434591@163.com (Q.Z.); 15097136110@163.com (D.G.); 18147119801@163.com (N.Q.)

**Keywords:** *Pseudomonas chlororaphis*-like, *Rhizoctonia solani*, cotton damping-off, RNA-seq, metabolomics, phenylpropanoid-associated defense

## Abstract

Cotton damping-off caused by *Rhizoctonia solani* restricts seedling establishment, and locally adapted biocontrol isolates are potential components of sustainable disease management. This study evaluated *Pseudomonas chlororaphis*-like isolate M6 and tested whether molecular profiles in M6-protected plants differed from those in Pathogen-infected plants in directions that moved toward Healthy Control levels. Culturable bacteria from root-associated soils were screened against *R. solani* by dual-culture assays, and M6 was evaluated in a greenhouse cotton damping-off assay. RNA sequencing (RNA-seq) and untargeted liquid chromatography–mass spectrometry (LC–MS) data from Healthy Control, Pathogen-infected, and M6-protected plants at 0 and 7 d were analyzed using the Pathogen-infected versus Healthy Control and M6-protected versus Pathogen-infected contrasts. M6 produced the highest inhibition among the candidate isolates (81.9%). Under greenhouse conditions, the M6-protected treatment had lower disease incidence (22.2% versus 88.9%) and disease index (16.4 versus 76.8) than the Pathogen-infected treatment. At 7 d, the primary false-discovery-rate-controlled analysis identified 4457 transcripts and 1620 LC–MS features for which the change between M6-protected and Pathogen-infected plants was opposite to the pathogen-associated change and the M6-protected group mean was closer to the Healthy Control mean. Additional nominal-*p*-value-only results were retained as exploratory. The two contrasts involved redox regulation, phenylpropanoid/benzenoid-related metabolism, hormone/defense signaling, protein synthesis and central metabolism. Antioxidant enzyme and quantitative reverse transcription PCR (qRT-PCR) data provided independent support for selected redox- and defense-associated differences. Because pathogen biomass, bacterial colonization and antagonism-deficient mutants were not assessed, the molecular profiles are interpreted as association-based features of the protected state rather than evidence that host recovery caused disease reduction. Most LC–MS annotations are putative and were not confirmed with authentic standards.

## 1. Introduction

Cotton damping-off caused by *Rhizoctonia solani* is an important seedling-stage disease in cotton production [1,2]. Disease severity reflects pathogen pressure, host developmental and physiological status, and environmental conditions; therefore, strong inhibition in an agar assay cannot be assumed to predict the dominant mode of action in planta [3]. A mechanistic evaluation of a candidate biocontrol isolate should distinguish directly observed disease reduction from molecular signatures that may arise secondarily because pathogen pressure or tissue injury has decreased.

The rhizosphere microbiome provides an ecological reservoir for such functional microorganisms [4,5]. Plant-derived resources, soil nutrient gradients and microbe–microbe interactions act as filters that shape microbial assembly and affect the probability of recovering beneficial taxa [6,7,8]. Disease-suppressive microbiomes are increasingly viewed as emergent systems in which microbial competition, antibiosis, resource partitioning and host-mediated immune modulation jointly contribute to disease suppression [9,10,11,12]. However, many studies still stop either at community profiling without functional validation or at strain screening without testing host-response mechanisms. This gap limits our ability to connect indigenous microbial resources with reproducible disease control.

Fluorescent *Pseudomonas* species are among the best-characterized groups of plant-associated biocontrol bacteria. Their activity may involve phenazines, pyrrolnitrin and other antimicrobial metabolites, siderophore-mediated competition, niche exclusion, motility and biofilm-related traits, as well as priming or modulation of plant immune responses [13,14,15,16,17,18,19,20,21,22]. *Pseudomonas chlororaphis* strains are already known for phenazine-associated antagonism and biocontrol; accordingly, the novelty of the present study is not a claim that M6 represents a previously unknown antagonistic mode. Rather, the study links isolate screening and greenhouse protection with an analysis that first defines the pathogen-associated disturbance and then evaluates whether the protected state shifts in the opposite direction. The relative contributions of direct antagonism, reduced pathogen biomass, and host-mediated processes cannot be resolved without pathogen quantification, colonization measurements, or bacterial functional mutants.

At the host level, redox regulation and phenylpropanoid/cell-wall-associated responses are relevant to *R. solani* challenge. Reactive oxygen species (ROS) function as immune signals but can also damage membranes, proteins, and cell walls, requiring regulation by superoxide dismutase (SOD), peroxidase (POD), catalase (CAT), and related systems [23,24,25]. Phenylpropanoid metabolism contributes precursors for phenolic compounds and lignin-associated polymers involved in cell-wall reinforcement [26,27,28]. Cotton studies have connected resistance-associated responses with ROS homeostasis, phenylpropanoid metabolism, and cell-wall defense [2,29,30]. Nevertheless, changes in these pathways are not uniquely diagnostic of resistance and may also reflect differences in pathogen burden or tissue damage.

Multi-omics analysis can describe molecular differences among treatment groups, but terminology must not imply causality. Standard differential expression and differential abundance analyses identify responsive features without determining whether a treatment directly repairs pathogen-induced injury. We therefore analyzed the three treatment groups at each sampling time without assigning a name to the design. First, Pathogen-infected plants were compared with Healthy Control plants to characterize the pathogen-associated difference. Second, M6-protected plants were compared with Pathogen-infected plants. A transcript or LC–MS feature was considered to move partially toward the Healthy Control level when the two differences had opposite signs, and the mean abundance in the M6-protected group was closer to the Healthy Control mean than the Pathogen-infected mean. This operational criterion describes the observed group differences and does not establish biological repair or causality.

In this study, we isolated indigenous culturable bacteria, screened them against *R. solani*, and selected isolate M6 for greenhouse validation in a cotton damping-off system because it showed the strongest antagonistic activity among the candidate isolates. We then integrated RNA-seq, untargeted metabolomics, RNA-metabolite module analysis, antioxidant enzyme assays, and qRT-PCR. We tested the hypothesis that reduced damping-off in M6-protected plants is associated with partial recovery of pathogen-disrupted host states. Throughout the study, we distinguished experimentally supported effects from association-based mechanistic interpretations.

## 2. Results

### 2.1. Screening Identifies M6 as a Highly Active Pseudomonas chlororaphis-like Antagonistic Isolate Against Rhizoctonia solani

**Figure 1:**
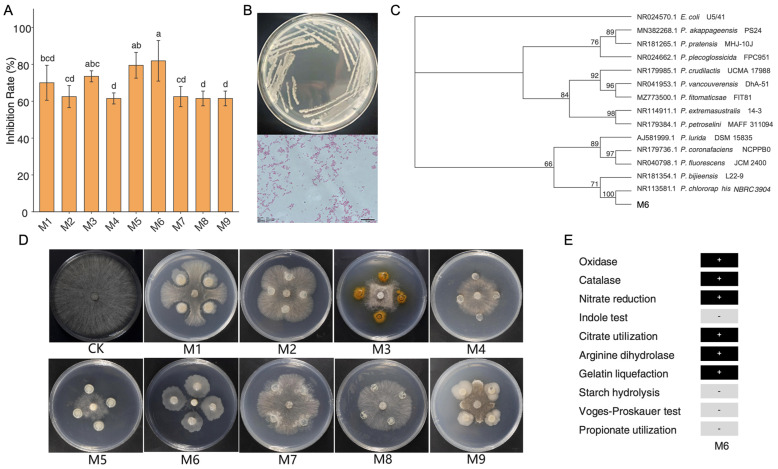
Screening, identification and characterization of the antagonistic isolate M6. (**A**) Inhibition rates of nine candidate bacterial isolates against *Rhizoctonia solani*. Different lowercase letters indicate significant differences among isolates (*p* < 0.05). (**B**) Colony morphology (upper) and Gram-stained cells (lower) of isolate M6. Scale bar = 100 μm. (**C**) 16S rRNA gene-based phylogenetic analysis showing the placement of isolate M6 within the *Pseudomonas chlororaphis* group. The analysis is not interpreted as definitive species-level identification. (**D**) Representative dual-culture assays showing the antagonistic activities of the nine candidate isolates against *R. solani*. CK, *R. solani* cultured without bacterial inoculation. (**E**) Physiological and biochemical characteristics of isolate M6. Positive (+) and negative (−) reactions indicate the presence and absence of the corresponding biochemical characteristics, respectively.

A total of 126 culturable bacterial isolates were recovered from root-associated soils and screened for antagonistic activity against *R. solani*. Nine isolates showing relatively strong inhibition were selected for comparative screening (Figure 1A,D). Among them, M6 produced the strongest inhibition of fungal growth, with an inhibition rate of 81.9%, significantly higher than most other candidate isolates (Figure 1A). This result identified M6 as the priority *P. chlororaphis*-like isolate for downstream greenhouse validation and host-response analysis.

M6 formed milky-white colonies and showed short rod-shaped cells after staining (Figure 1B). Physiological and biochemical tests showed positive reactions for oxidase, catalase, nitrate reduction, citrate utilization, arginine dihydrolase, and gelatin liquefaction, and negative reactions for indole production, starch hydrolysis, Voges-Proskauer reaction, and propionate utilization (Figure 1E). The 16S rRNA sequence placed M6 near *Pseudomonas chlororaphis* reference sequences; however, 16S rRNA and phenotypic traits do not provide reliable species-level resolution within Pseudomonas [31]. M6 is therefore described throughout as a *P. chlororaphis*-like isolate. Definitive assignment requires multilocus sequence analysis with authenticated type strains or genome-based average nucleotide identity/digital DNA–DNA hybridization.

### 2.2. M6 Treatment Is Associated with Reduced Cotton Damping-Off and Partial Retention of Seedling Growth Under Pathogen Challenge

**Figure 2:**
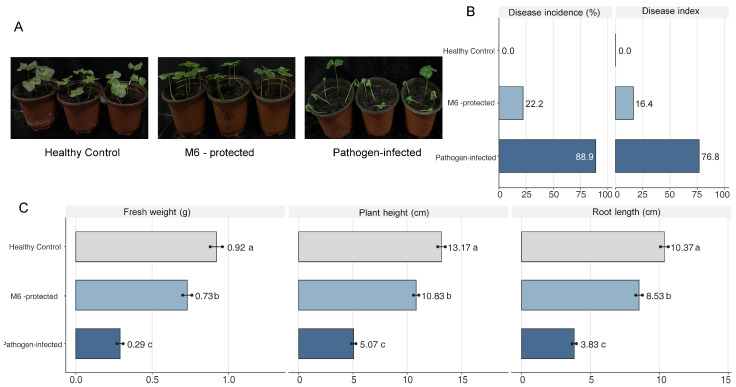
Greenhouse validation of M6 protection against cotton damping-off. (**A**) Representative seedling phenotypes under Healthy Control, M6-protected, and Pathogen-infected treatments. (**B**) Disease incidence and disease index. (**C**) Fresh weight, plant height, and root length. Values are treatment means from three biological replicate pots (*n* = 3); error bars show variation among biological replicates. Different lowercase letters indicate significant differences at *p* < 0.05. The pot was treated as a biological replicate.

The greenhouse assay confirmed that the activity of M6 was retained in planta. *R. solani* infection caused severe damping-off symptoms, whereas M6-protected seedlings showed visibly reduced disease severity (Figure 2A). Disease incidence reached 88.9% in Pathogen-infected plants and decreased to 22.2% in the M6-protected treatment. Disease index decreased from 76.8 to 16.4 (Figure 2B). Thus, M6 reduced disease incidence by approximately 75.0% and disease index by approximately 78.6% relative to the Pathogen-infected treatment.

Growth measurements were consistent with the disease assessment (Figure 2C). *R. solani* reduced fresh weight, plant height, and root length to 0.29 g, 5.07 cm, and 3.83 cm, respectively, compared with 0.92 g, 13.17 cm, and 10.37 cm in Healthy Control seedlings. M6-protected seedlings retained substantially higher values under pathogen challenge, with fresh weight, plant height, and root length reaching 0.73 g, 10.83 cm, and 8.53 cm, respectively. These results indicate that M6 treatment was associated with reduced cotton damping-off and alleviation of pathogen-induced seedling growth inhibition. Because an M6-only treatment was not included, these data should not be interpreted as evidence for M6-driven growth promotion in the absence of pathogen stress.

### 2.3. Rhizoctonia solani Infection Is Associated with Extensive Transcriptomic and Metabolic Changes in Cotton Roots

To establish the molecular background associated with pathogen infection, we first compared the Pathogen-infected and Healthy Control treatments at 7 d. A total of 22,065 genes (10,563 increased and 11,502 decreased) were differentially expressed (|log_2_FC| ≥ 0.5, FDR < 0.05). KEGG enrichment indicated that these genes were mainly associated with ribosome, secondary metabolite biosynthesis, amino acid biosynthesis, phenylpropanoid biosynthesis, oxidative phosphorylation, and carbon metabolism (Supplementary Data S1).

Untargeted LC–MS analysis identified 6782 differential features (2470 increased and 4312 decreased) between the Pathogen-infected and Healthy Control treatments (|log_2_FC| ≥ 0.5, FDR < 0.05). Putative annotations suggested that these features were mainly assigned to lipid and lipid-like molecules, organoheterocyclic compounds, phenylpropanoids/polyketides, benzenoids and organic acids. Because most metabolite identities were assigned by spectral-library matching rather than authentic standards, these annotations are considered putative (Supplementary Data S2).

These pathogen-associated transcriptomic and metabolic differences provided the reference for the subsequent comparison between the M6+Rs and Pathogen-infected treatments. Owing to the absence of pathogen biomass quantification, the observed molecular differences are interpreted as treatment-associated changes rather than evidence of direct host recovery.

### 2.4. Transcriptomic Responses of Cotton Roots to R. solani Infection and M6 Treatment

**Figure 3:**
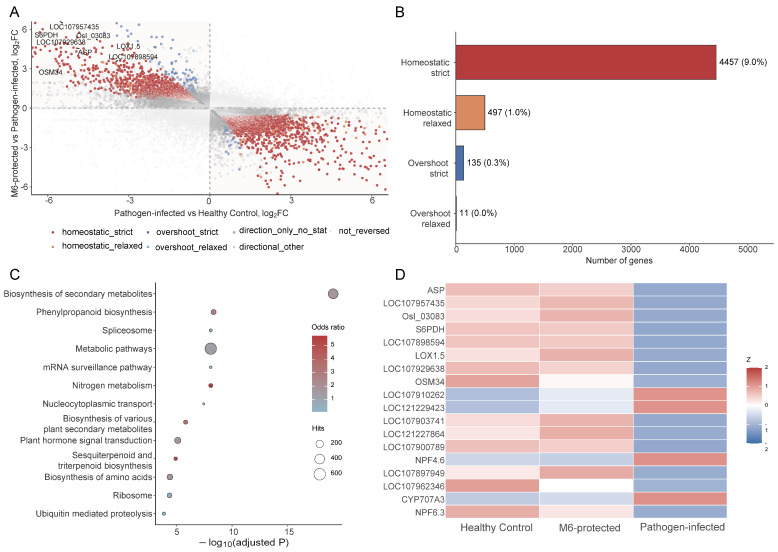
Transcriptional differences among Healthy Control, Pathogen-infected and M6-protected roots at 7 d. (**A**) Relationship between the Pathogen-infected versus Healthy Control and M6-protected versus Pathogen-infected log_2_ fold changes. (**B**) Numbers of transcripts meeting the FDR-controlled criteria for opposite-direction changes toward the Healthy Control mean, corresponding exploratory nominal-p results, and transcripts that changed beyond the Healthy Control mean. The primary inferential set required |log2 fold change| ≥ 0.5 and FDR < 0.05 in both contrasts. (**C**) FDR-significant pathways represented among the primary transcript set. (**D**) Selected transcript profiles across the three treatments. Each treatment contained three biological replicates.

Each transcript was evaluated using the Pathogen-infected versus Healthy Control and M6-protected versus Pathogen-infected contrasts. The primary analysis required |log_2_ fold change| ≥ 0.5 and Benjamini–Hochberg FDR < 0.05 in both contrasts, opposite signs for the two differences, and a group mean in M6-protected plants that was closer to the Healthy Control mean than the Pathogen-infected mean. A total of 4457 transcripts met these criteria (Figure 3B). A further 497 transcripts met the directional and effect-size criteria but achieved only nominal *p* < 0.05 in both contrasts and were retained as exploratory results. In addition, 135 FDR-controlled and 11 exploratory transcripts changed in the opposite direction but extended beyond the Healthy Control group mean.

The 4457 FDR-controlled transcripts were concentrated in the two opposite-direction quadrants (Figure 3A): pathogen-associated increases were accompanied by lower abundance in M6-protected than in Pathogen-infected plants, whereas pathogen-associated decreases were accompanied by higher abundance. For these transcripts, the M6-protected group mean was closer to the Healthy Control mean. This pattern does not establish that M6 directly repaired host processes, because it could also arise if M6 reduced pathogen burden and consequently reduced secondary host stress.

The 4457-transcript set was enriched in ribosome-related processes, amino acid biosynthesis, nitrogen metabolism, ubiquitin-mediated proteolysis, plant hormone signal transduction, secondary metabolism, and phenylpropanoid biosynthesis (Figure 3C). The selected transcripts in Figure 3D illustrate both directions of change at the treatment-mean level. Enrichment results describe processes represented among transcripts whose abundance in M6-protected plants was closer to Healthy Control levels and are not interpreted as proof that any pathway caused disease reduction.

### 2.5. Metabolic Responses of Cotton Roots to R. solani Infection and M6 Treatment

**Figure 4:**
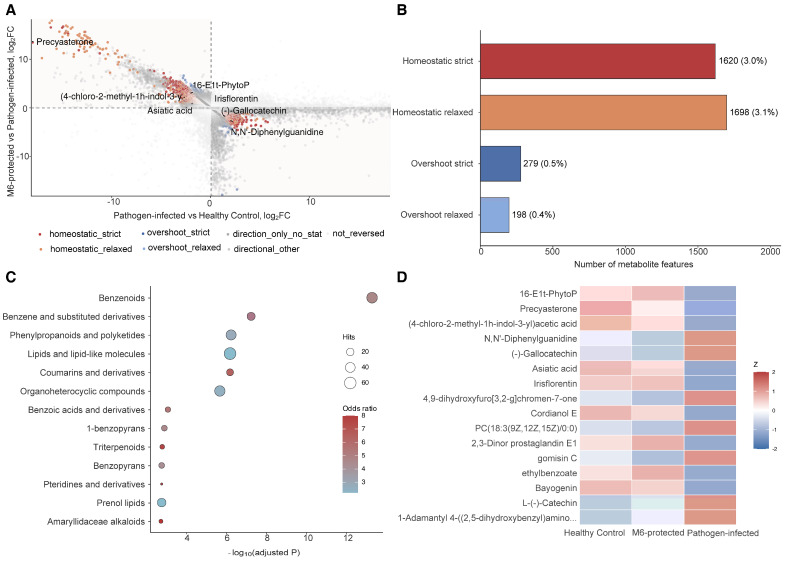
LC-MS feature differences among Healthy Control, Pathogen-infected and M6-protected roots at 7 d. (**A**) Relationship between the Pathogen-infected versus Healthy Control and M6-protected versus Pathogen-infected log_2_ fold changes. (**B**) Numbers of features meeting the FDR-controlled criteria for opposite-direction changes toward the Healthy Control mean, corresponding exploratory nominal-p results, and features that changed beyond the Healthy Control mean. (**C**) FDR-significant chemical classes represented among the primary feature set. (**D**) Selected putatively annotated features across the three treatments. Most annotations are database-supported and were not confirmed with authentic standards; feature-specific evidence and confidence levels are reported in Supplementary Data. Each treatment contained three biological replicates.

The same criteria were applied to LC–MS features. The primary FDR-controlled set contained 1620 features, representing 3.0% of the tested features, for which the two contrasts had opposite signs and the M6-protected group means were closer to the Healthy Control mean (Figure 4B). A further 1698 features met the effect-size and directional criteria but only nominal *p* < 0.05 in both contrasts and were retained as exploratory results. In addition, 279 FDR-controlled and 198 exploratory features changed in the opposite direction but extended beyond the Healthy Control group mean. The exploratory set was not used as the principal basis for mechanistic claims.

At the chemical-class level, the 1620 FDR-controlled features were overrepresented among benzenoids, phenylpropanoids/polyketides, lipids and lipid-like molecules, coumarin-related features, organoheterocyclic compounds, and benzoic-acid-related classes (Figure 4C). Figure 4D shows selected database-matched features whose group means in M6-protected plants were closer to Healthy Control levels than those in Pathogen-infected plants. In accordance with Metabolomics Standards Initiative reporting principles [32], these names are treated as putative annotations unless an authentic standard analyzed under the same conditions is documented; none is presented as a level-1 identification solely on the basis of database matching.

### 2.6. Annotation-Based Relationships Between Transcript and LC–MS Feature Groups

Taken together, the transcriptomic and LC–MS analyses showed that *R. solani* infection was associated with coordinated changes in nutrient transport, hormone-related regulation, lipid- and redox-associated processes, osmotic and defense responses, central metabolism, and benzenoid/phenylpropanoid-related chemical features. These pathogen-associated changes were subsequently used as the reference for evaluating whether molecular differences between M6-protected and Pathogen-infected plants occurred in the opposite direction and shifted selected features toward the Healthy Control state.

**Figure 5:**
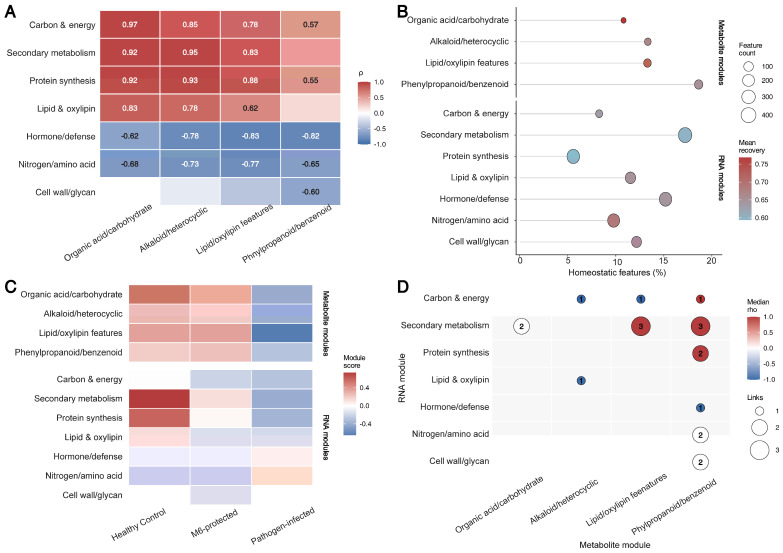
Descriptive RNA-LC-MS feature-group associations at 7 d. (**A**) Spearman correlation matrix between annotation-defined RNA and LC-MS feature-group scores across nine samples; group-level *p*-values were adjusted using the Benjamini–Hochberg method. (**B**) Numbers and proportions of transcripts and LC-MS features meeting the primary FDR-controlled criteria in each group. (**C**) Treatment-level group-score heatmap. (**D**) Exploratory feature-level associations summarized by group pair; these links were not interpreted as direct regulation and were not used as independent causal evidence.

To summarize the cross-omics structure, the FDR-controlled transcripts and LC–MS features whose M6-protected means were closer to Healthy Control levels were assigned to annotation-defined functional groups rather than inferred as regulatory modules. Seven RNA groups and four LC–MS feature groups were retained for the main analysis (Figure 5). Across the nine day-7 samples, RNA-group and LC–MS-group scores showed positive associations between carbon/energy transcripts and organic-acid/carbohydrate-like features and between secondary-metabolism transcripts and alkaloid/heterocyclic-like, or lipid/oxylipin-like features. Hormone/defense and nitrogen/amino-acid RNA scores were negatively associated with several LC–MS feature-group scores. Because only nine samples were available, these correlations are interpreted as descriptive coordination and not as evidence of regulatory coupling.

The RNA functional groups contained 417 secondary-metabolism, 346 protein-synthesis, 332 hormone/defense, 298 nitrogen/amino-acid, 190 lipid/oxylipin, 162 cell-wall/glycan, and 55 carbon/energy transcripts meeting the FDR-controlled criteria (Figure 5B). The corresponding LC–MS groups contained 95 phenylpropanoid/benzenoid-like, 69 lipid/oxylipin-like, 46 alkaloid/heterocyclic-like, and 32 organic-acid/carbohydrate-like features. Group scores placed M6-protected samples between, or closer to, Healthy Control and Pathogen-infected states depending on the functional group (Figure 5C). Group-level correlation *p*-values were adjusted by the Benjamini–Hochberg method. Feature-level links were filtered by |Spearman ρ| ≥ 0.80 and nominal *p* ≤ 0.01 and are presented only as hypothesis-generating associations, not as direct gene-to-metabolite relationships (Figure 5D).

### 2.7. Antioxidant Enzyme and qRT-PCR Validation Support M6-Associated Defense Recovery

**Figure 6:**
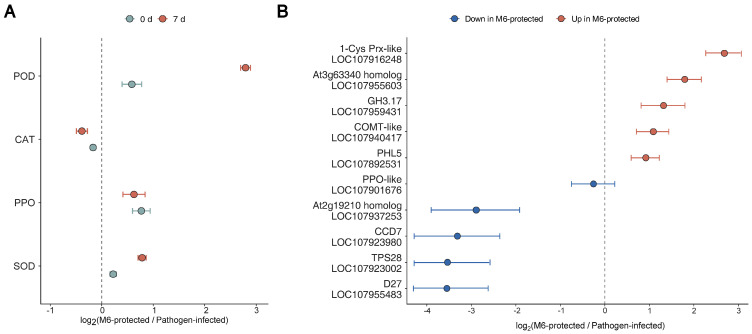
Antioxidant enzyme and qRT-PCR validation of M6-associated defense recovery. (**A**) log_2_ ratios of antioxidant enzyme activities in M6-protected versus Pathogen-infected plants at 0 and 7 d. (**B**) log_2_ ratios of selected qRT-PCR genes in M6-protected versus Pathogen-infected plants at 7 d.

We next examined whether the multi-omics recovery model was supported by independent antioxidant enzyme and qRT-PCR validation (Figure 6). Because POD, CAT, PPO, and SOD differed strongly in absolute activity ranges, enzyme responses were visualized as log_2_ ratios of M6-protected to Pathogen-infected plants. This effect-size representation allowed direct comparison of M6-associated shifts across enzymes and time points. At 7 d, POD showed the strongest positive effect, while SOD and PPO also increased in M6-protected plants relative to Pathogen-infected plants. CAT showed a negative effect at 7 d. These patterns indicate selective antioxidant adjustment rather than uniform induction of all measured enzymes.

qRT-PCR analysis at 7 d supported selected RNA-seq patterns (Figure 6B). Genes annotated as 1-Cys Prx-like, At3g63340 homolog, GH3.17, COMT-like, and PHL5 showed higher transcript abundance in M6-protected plants, whereas D27, TPS28, CCD7, and an At2g19210 homolog showed lower abundance. This bidirectional pattern is consistent with the recovery framework, in which M6 protection is associated with rebalancing of pathogen-disrupted transcriptional states rather than uniform activation of all tested defense-related genes.

## 3. Discussion

### 3.1. Contribution and Scope of the M6 Study

This study links the screening of an indigenous *Pseudomonas chlororaphis*-like isolate with a lower-severity greenhouse phenotype and multi-omics characterization of the protected host state. *P. chlororaphis* strains are already established biocontrol organisms, and the present data do not identify a novel phenazine pathway or demonstrate a strain-specific antagonistic metabolite. The contribution is instead the explicit analysis of the pathogen-associated difference before the corresponding comparison between M6-protected and Pathogen-infected plants across transcript, LC–MS feature, and bio-chemical datasets. This approach provides more biological context than a list of M6-responsive genes or features while remaining association-based.

The 81.9% inhibition observed in dual culture establishes that M6 can restrict *R. solani* growth under the assay conditions, and the greenhouse experiment establishes an association with lower disease incidence and severity. Neither result demonstrates that in vitro antagonism is the primary mechanism in planta. Soil properties, bacterial establishment, pathogen biomass and plant condition can all influence disease outcome. Consequently, plate antagonism is treated as one plausible component of the M6 phenotype rather than as a causal explanation for greenhouse protection [3,13,14,15,16,17,18,19,20,21,22].

### 3.2. Pathogen-Associated Perturbation and Molecular Profiles of M6-Protected Plants

Characterizing the Pathogen-infected versus Healthy Control contrast before the M6-protected versus Pathogen-infected contrast materially changes the interpretation. *R. solani* was associated with broad perturbation of redox, protein-synthesis, nitro-gen/amino-acid, hormone/defense, secondary-metabolism, cell-wall, and central-metabolism features. Cotton proteomic work on *R. solani* infection similarly implicated ROS homeostasis and phenylpropanoid biosynthesis [2]. We then identified transcripts and LC–MS features for which the difference between M6-protected and Pathogen-infected plants was opposite to the pathogen-associated difference and moved the group mean toward the Healthy Control level. This analysis advances beyond isolated DEG or differential-feature lists by making the direction of the disease-associated difference explicit; however, it does not resolve whether the observed pattern was driven by lower fungal biomass, direct M6 signaling, or both.

### 3.3. Redox and Phenylpropanoid-Associated Signatures

The enzyme data provide a physiological anchor for the redox-associated transcript patterns. ROS participate in pathogen signaling but can also amplify tissue injury, and resistance is therefore associated with regulated spatial and temporal redox dynamics rather than maximal activity of every antioxidant enzyme [23,24,25]. The large POD difference and higher SOD activity in M6-protected roots, together with lower CAT activity, indicate selective reconfiguration of the measured enzyme system. *R. solani* resistance screening in potato has likewise shown that disease severity covaries with CAT, peroxidase, and PPO activities, illustrating that enzyme profiles can serve as physiological indicators without, by themselves, identifying causal defense mechanisms [33].

Phenylpropanoid and cell-wall-associated features were represented across the RNA-seq, LC–MS feature, and qRT-PCR datasets. Phenylpropanoid metabolism supplies precursors for phenolics and lignin-associated polymers that can reinforce cell walls [26,27,28]. Cotton studies have provided direct evidence that cell-wall defense genes and auxin-linked regulatory processes contribute to fungal disease resistance [29], while bio-control-associated cotton transcriptomics has implicated ROS, flavonoid, and phenylpropanoid pathways [30]. Our data are consistent with this literature, but they do not include lignin staining, cell-wall compositional measurements, or authentic-standard quantification of phenolic compounds. The appropriate conclusion is therefore a phenylpropanoid/cell-wall-associated signature, not demonstrated lignification.

### 3.4. Cross-Omics Coordination and Targeted Validation

The annotation-defined RNA–LC–MS feature groups summarized coordinated changes that were not evident from a single marker. Positive and negative correlations among group scores may reflect altered substrate supply, defense allocation, or reduced disease pressure. With only nine day-7 samples and annotation-based feature grouping, these relationships are exploratory. The qRT-PCR and enzyme assays strengthen confidence that selected redox, phenylpropanoid, hormone, and signaling changes were -reproducible across analytical platforms, but they do not convert the cross-omics correlations into regulatory links.

A conservative interpretation is that the molecular profile of M6-protected seedlings reflects a combination of reduced pathogen pressure, reduced tissue injury and/or altered host responses. Pathogen biomass was not quantified, and the experiment therefore cannot distinguish reduced pathogen load from host-mediated recovery. M6 colonization dynamics, secondary-metabolite production, and antagonism-deficient mutants were also not assessed, and species-level identity remains unresolved without multilocus or genome-based analysis [31]. In addition, the study was conducted under greenhouse conditions and does not establish field performance. These limitations constrain the mechanistic and translational claims.

The protected-state molecular differences nevertheless define testable next steps: quantitative PCR of *R. solani* biomass, genome-resolved characterization of M6, root-colonization assays, targeted analysis of bacterial antimicrobial metabolites, ROS imaging, lignin/cell-wall measurements, and perturbation of prioritized host genes. These experiments are required to determine whether the observed differences are consequences of reduced disease, contributors to protection, or both.

### 3.5. Limitations and Conservative Working Model of the M6-Associated Protected State

**Figure 7:**
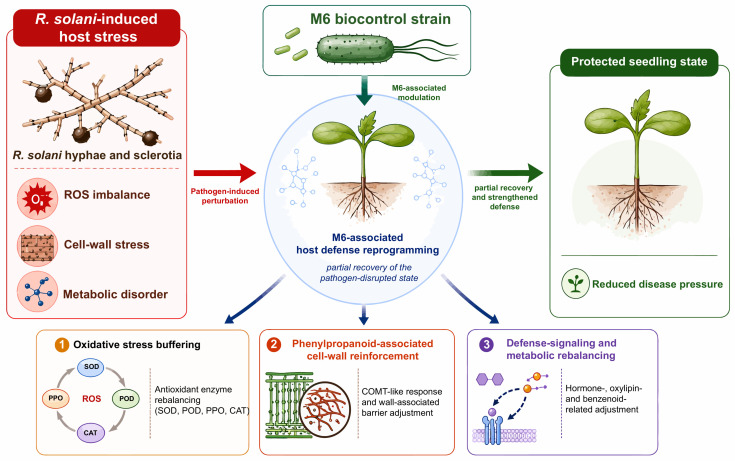
Conservative working model of M6-associated host-state recovery against Rhizoctonia solani. The model summarizes a pathogen-disturbed host state, M6-associated modulation, partial defense recovery, and reduced disease pressure. The proposed outputs include antioxidant enzyme rebalancing, phenylpropanoid-associated cell-wall responses, and defense-metabolic rebalancing. The model should be interpreted as association-based and hypothesis-generating rather than as causal proof of host-mediated repair.

Figure 7 summarizes an association-based working model. *R. solani* infection is represented as a perturbed state involving oxidative, cell-wall, and metabolic changes. M6 treatment is linked to lower disease severity and to partial movement of redox-, phenylpropanoid/cell-wall-, and defense-metabolic-associated measurements toward Healthy Control levels. The model deliberately leaves open two non-exclusive explanations: M6 may lower pathogen pressure, thereby reducing secondary host disturbance, and/or may alter host responses directly. The current experiment cannot estimate the relative contribution of these routes.

## 4. Materials and Methods

We used a GenAI tool (ChatGPT-5.5) solely for language polishing and grammar correction during the preparation of this manuscript. No AI tools were used for data collection, or analysis, interpretation.

### 4.1. Pathogen Culture, Bacterial Isolation and Antagonistic Screening

*Rhizoctonia solani* was maintained on potato dextrose agar (PDA) at 25 °C and subcultured before antagonistic assays. Actively growing 5 mm mycelial plugs were taken from the colony margin of 3-day-old PDA cultures and used as pathogen inoculum.

Root-associated soil samples were collected with sterile tools and transported to the laboratory under cooled conditions. For bacterial isolation, 10 g of fresh soil was suspended in 90 mL sterile saline, shaken at 180 rpm for 30 min, and serially diluted from 10^−1^ to 10^−6^. Aliquots of 100 μL from appropriate dilutions were spread onto Luria–Bertani agar plates and incubated at 28 °C for 24–48 h. Colonies with distinct morphologies were picked and purified by repeated streaking until single-colony cultures were obtained. Purified isolates were maintained on LB agar for short-term use and stored in LB broth supplemented with 20% glycerol at −80 °C.

Primary antagonistic screening was performed using a dual-culture assay. A fresh *R. solani* mycelial plug was placed in the center of a PDA plate, and bacterial isolates were inoculated at equal distances from the plug. Plates inoculated with *R. solani* alone served as pathogen controls. Cultures were incubated at 25 °C until the fungal colony in the control approached the plate margin. Fungal radial growth was measured in the direction of bacterial growth and compared with the control. The inhibition rate was calculated as inhibition rate (%) = [(Rcontrol − Rtreatment)/Rcontrol] × 100, where Rcontrol and Rtreatment represent the fungal radial growth in the control and dual-culture plates, respectively [3]. Isolates with high inhibition rates were retained for secondary screening and visualization.

### 4.2. Strain Identification and Physiological Characterization

Genomic DNA of M6 was extracted from pure culture. The 16S rRNA gene was amplified using primers 27F and 1492R and sequenced. Taxonomic placement was evaluated by BLAST v2.17.0 and phylogenetic comparison with *Pseudomonas* reference sequences. Colony morphology, cell morphology, and physiological/biochemical traits were used for phenotypic characterization but not for definitive species assignment. Because 16S rRNA provides insufficient species-level resolution for many environmental *Pseudomonas* isolates [31], M6 is described as a *P. chlororaphis*-like isolate. Multilocus sequence analysis using authenticated type-strain references or whole-genome ANI/dDDH is required for species-level confirmation.

### 4.3. Greenhouse Biocontrol Experiment

Cotton seeds were surface-sterilized, rinsed thoroughly with sterile distilled water, and sown in sterilized soil under greenhouse conditions at 25 ± 2 °C. Three treatments were established: Healthy Control, Pathogen-infected, and M6-protected. The Healthy Control treatment received sterile water only. The Pathogen-infected treatment was inoculated with *R. solani*. The M6-protected treatment received M6 suspension followed by *R. solani* inoculation. Each treatment contained three biological replicates, and pots were arranged using a randomized design.

M6 was cultured in LB broth to logarithmic phase, centrifuged, and resuspended to approximately 1 × 10^8^ CFU mL^−1^. Bacterial suspension was applied by root irrigation at the seedling stage. *R. solani* was inoculated using actively growing PDA mycelial plugs after M6 application. Disease incidence was calculated as the percentage of diseased seedlings. Disease index was calculated from categorical symptom ratings using the standard formula: disease index = Σ(class value × number of plants in that class)/(maximum class value × total number of plants) × 100 [34,35]. Seedling fresh weight, plant height, and root length were measured at 7 d after pathogen inoculation.

### 4.4. Construction of the Transcriptomic and Metabolomic Datasets

Transcriptomic and metabolomic analyses included samples collected at 0 and 7 d from the Control, Rs, and M6+Rs treatments. Each treatment contained three biological replicates at each sampling time, resulting in 18 samples for each omics dataset. Only LC–MS features detected and quantified across all three treatments at the corresponding sampling time were retained for comparative analysis. The final datasets contained 49,815 expressed genes and 54,362 LC–MS features.

### 4.5. RNA-Seq Processing and Differential Expression Analysis

Total RNA was extracted from cotton roots using TRIzol reagent (Sigma-Aldrich, USA). RNA integrity and concentration were assessed before library preparation. Libraries were sequenced on an Illumina NovaSeq platform in paired-end mode. Raw reads were quality-controlled using fastp v0.22.0, and clean reads were aligned to the Gossypium hirsutum TM-1 reference genome using HISAT2 v2.1.0. Gene-level counts were generated using HTSeq v0.9.1. Differential expression was analyzed in R, using DESeq2 v1.38.3 with raw counts and the Benjamini–Hochberg false discovery rate (FDR) correction [36]. Pairwise contrasts were performed separately for each time point: Pathogen-infected versus Healthy Control, M6-protected versus Pathogen-infected, and M6-protected versus Healthy Control. Reported differential effects include log_2_ fold changes and adjusted *p*-values. Sample-level PCA and expression distribution plots were used for quality assessment; no explicit batch term was included because the complete-3 RNA-seq samples were processed as a single balanced experiment.

### 4.6. Metabolite Extraction, LC-MS Profiling and Differential Abundance Analysis

Root tissue powder was extracted with precooled methanol:acetonitrile:water (2:2:1, *v*/*v*/*v*) containing 2-chlorophenylalanine as an internal standard. Extracts were homogenized, sonicated, incubated at low temperature and centrifuged, and supernatants were filtered before LC-MS analysis. Pooled quality-control (QC) samples were prepared to monitor instrument stability during acquisition.

Untargeted profiling was performed by ultra-high-performance liquid chromatography coupled to a high-resolution Orbitrap mass spectrometer in positive and negative ion modes. Raw data were processed with MS-DIAL v4.9.221218 for peak detection, alignment, missing-value handling, and normalization [37]. The MS1 and MS2 mass tolerances were 0.01 and 0.05 Da, respectively. Feature annotations were generated from accurate mass and available MS/MS spectral matches against mzCloud, HMDB, LIPID MAPS, and the service-provider spectral library. Following Metabolomics Standards Initiative principles [32], no feature is reported as a level-1 identification unless retention time and spectral properties were matched to an authentic standard analyzed under the same conditions. Spectral-library matches without same-run authentic standards are reported as putative level-2 annotations; class-level assignments are reported as level 3; unmatched signals remain *m*/*z*–retention-time features. Differential abundance was analyzed from log_2_-transformed, median-normalized intensities using empirical-Bayes linear modeling in limma [38], with Benjamini–Hochberg FDR correction. Raw files, processed matrices, QC metadata, and feature-specific annotation evidence are required components of the public data release.

### 4.7. Identification of Transcripts and LC–MS Features Showing Opposite-Direction Changes

For both RNA-seq and metabolomic data, the pathogen-associated perturbation was defined as the log_2_ fold change in Pathogen-infected versus Healthy Control, and the M6-associated counter-shift was defined as the log_2_ fold change in M6-protected versus Pathogen-infected. A feature was considered directionally reversed when the two log_2_ fold changes had opposite signs. To avoid classifying very small fluctuations as recovery, features required absolute log_2_ fold change values of at least 0.5 in both contrasts. Strict recovery required FDR < 0.05 in both contrasts; relaxed recovery required nominal *p* < 0.05 in both contrasts. Direction-only features lacking statistical support were not used as primary recovery candidates.

A recovery index was calculated as RI = 1 − |M6-protected − Healthy Control|/|Pathogen-infected − Healthy Control| using group means at 7 d. Features with statistically supported directional reversal and positive movement toward the Healthy Control state were classified as homeostatic recovery features. Features showing statistically supported counter-directional movement beyond the Healthy Control state were classified as overshoot reversal features. Functional enrichment for recovery genes and metabolite classes was tested against the corresponding tested feature background using Fisher’s exact tests followed by Benjamini–Hochberg FDR correction [39].

### 4.8. RNA-Metabolome Module Integration

Homeostatic recovery features were assigned to biologically interpretable modules using annotation-based keyword rules. RNA modules included carbon and energy metabolism, secondary metabolism, protein synthesis, lipid and oxylipin metabolism, hormone/defense signaling, nitrogen/amino acid metabolism, and cell wall/glycan remodeling. Metabolite modules included organic acid/carbohydrate-like features, alkaloid/heterocyclic-like features, lipid/oxylipin-like features, and phenylpropanoid/benzenoid-like features. Broad “other” modules were retained in supplementary summaries but excluded from the main figure.

For each retained module, a sample-level module score was calculated as the mean row-scaled abundance of homeostatic recovery features within that module. Spearman correlations between RNA module scores and metabolite module scores were calculated across the nine day-7 complete-3 samples. *p*-values were adjusted using the Benjamini–Hochberg method. Curated gene-metabolite associations were retained only as hypothesis-generating links when |rho| >= 0.80 and *p* <= 0.01, and only when both features belonged to interpretable retained modules. These associations were not interpreted as direct regulatory relationships.

### 4.9. Antioxidant Enzyme Assays

Fresh cotton root samples were homogenized on ice in precooled phosphate buffer and centrifuged at 12,000× *g* for 20 min at 4 °C. The supernatant was used as crude enzyme extract. Peroxidase (POD), catalase (CAT), polyphenol oxidase (PPO) and superoxide dismutase (SOD) activities were determined using commercial micro-assay kits according to the manufacturer’s instructions. Enzyme activities were expressed as U g^−1^ fresh weight. For visualization, enzyme effects were summarized as log_2_ ratios of M6-protected to Pathogen-infected plants, with approximate confidence intervals propagated from standard errors.

### 4.10. qRT-PCR Validation

Selected differentially expressed genes were validated by qRT-PCR. Total RNA was reverse-transcribed into cDNA, and amplification was performed using a SYBR Green-based system. Each 20 μL reaction contained 10 μL SYBR Premix Ex Taq, 0.4 μL each of forward and reverse primers, 2 μL cDNA template, and 7.2 μL sterile water. The amplification program consisted of 95 °C for 30 s, followed by 40 cycles of 95 °C for 5 s and 60 °C for 30 s. Actin (LOC107959437) was used as the internal reference gene. Relative transcript abundance was calculated using the 2^−ΔΔCt^ method [40]. Primer sequences are listed in Table 1.

### 4.11. Statistical Analysis

All statistical analyses for phenotypic, antagonistic, and biochemical data were performed in R. Data distribution and variance homogeneity were examined before group comparison. For normally distributed data with homogeneous variance, one-way ANOVA followed by Tukey’s HSD test was used. When assumptions were not met, Kruskal–Wallis tests followed by post hoc pairwise comparisons were used. Differences were considered significant at *p* < 0.05.

## 5. Conclusions

*Pseudomonas chlororaphis*-like isolate M6 showed strong in vitro antagonism and was associated with markedly lower cotton damping-off severity under greenhouse conditions. The Pathogen-infected versus Healthy Control contrast demonstrated that *R. solani* perturbed redox, defense, cell-wall, and central-metabolic states. FDR-controlled RNA-seq and LC–MS analyses subsequently identified counter-directional features in M6-protected roots, with consistent representation of redox and phenylpropanoid/benzenoid-related processes. qRT-PCR and enzyme activities supported selected components of this protected-state signature. The data do not determine whether these patterns arose from lower pathogen biomass, direct host modulation, or both. The study therefore identifies M6 as a candidate biocontrol isolate and provides association-based hypotheses for causal testing rather than a demonstrated antagonism–host-recovery mechanism.

## Figures and Tables

**Table 1:** Gene-specific primers used for qRT-PCR validation.

Validation Category	Reverse Primer (5′ to 3′)	Forward Primer (5′ to 3′)	Putative Annotation/Gene Name	Gene ID
Selected DEG	TGTGTCCTCCTGATAGAA	GAAGAGATGGAAGGGTTT	Candidate DEG	LOC107946327
Oxidative defense	AATACTCTCATCACCTTCAAC	TCTACCGCATAACCATCA	PPO-like/polyphenol oxidase-like	LOC107901676
Selected DEG	CATCTTGATTGTGTTGTT	CGAGTCTTGATTATGGTT	Candidate DEG	LOC107944640
Redox-related	CAATAGTGAGGAGTGGAA	AATAGGCTTGATGTGGAA	At3g63340 homolog/SOD-related candidate	LOC107955603
Selected DEG	ATGGTGATTATTGTGATGA	TCTTCTCCTATTCTGACA	Candidate DEG	LOC107943729
Phenylpropanoid-related	AAGAACCGACTTCAAGAA	AAGCATCCACAGATTAGG	COMT-like/caffeic acid 3-O-methyltransferase-like	LOC107940417
Selected DEG	AAGACACCACCACTAGATTAC	ACCGCAACTTGACAGATT	Candidate DEG	LOC107936045
Terpenoid-related	CACCTCCATATTGTTCTA	CGGCTGATGAATTATTAG	TPS28	LOC107923002
Carotenoid/strigolactone-related	GTTGCGAGATGAATAGAA	TACATCAACCATCATTACC	CCD7	LOC107923980
Carotenoid/strigolactone-related	GTTATCATTATGGACACTACTG	GAGGTTGAGGTTGAAGTT	D27	LOC107955483
Selected DEG	TTCGGTGATTGTGGAGTC	CCAGACCTGCTATCAAGTG	Candidate DEG	LOC107918485
Selected DEG	TGTTGTTGAATGCTTGAA	GCTTAGATTGTGGATTACC	At2g19210 homolog	LOC107937253
Selected DEG	ATAGAAGGTGAAGTATAAGAATG	CAGGTTGTGATTGAGTTG	Candidate DEG	LOC107941509
Redox-related	TGGATACAGAATGCTCAG	CAACTTAACATGGTGGAC	1-Cys peroxiredoxin-like	LOC107916248
Hormone-related	AGTATCTTCATTGTTGCTCTT	AACCTTGTGATGTCTCCTA	GH3.17	LOC107959431
Selected DEG	GGATAATGATGAACAAGAATC	AACAAGGAGAACCAACTA	Candidate DEG	LOC107886227
Selected DEG	GGATACAATCTCATAGACTCA	TGGTTCTTCTGGTTAATACA	Candidate DEG	LOC121219928
Selected DEG	TTGATGATGGTGGAGTTAT	TGGAGATGAAGAAGAGAAG	Candidate DEG	LOC107894076
Selected DEG	ACTCTCCACCACTTCAAT	GACTCACCACCTTATCCT	Candidate DEG	LOC107940910
Defense-related	GCAGTAAGAAGAAGTATCCA	TCATCATCATCCAATCCAAT	PHL5	LOC107892531
Reference gene	AATAAGAGATGGCTGGAA	CTGTTGAGAAGAACTATGAG	Actin	LOC107959437

## Data Availability

Raw RNA-seq reads have been deposited in the NCBI Sequence Read Archive under BioProject accession PRJNA1452184. Processed RNA-seq count matrices, differential-expression results, LC–MS feature matrices, annotation evidence and statistical results are provided as Supplementary Data S1 and S2.

## Supplementary Materials

The following supporting information can be downloaded at: https://www.mdpi.com/article/10.3390/plants15152305/s1. The raw RNA-seq data and raw metabolomics data are provided in Supplementary Data S1 and S2, respectively.

## Abbreviations

The following abbreviations are used in this manuscript:

CAT	catalase
COMT	caffeic acid 3-O-methyltransferase
DEG	differentially expressed gene
FDR	false discovery rate
LC-MS	liquid chromatography–mass spectrometry
PDA	potato dextrose agar
POD	peroxidase
PPO	polyphenol oxidase
ROS	reactive oxygen species
SOD	superoxide dismutase
